# Association of Smoking with Metabolic Volatile Organic Compounds in Exhaled Breath

**DOI:** 10.3390/ijms18112235

**Published:** 2017-10-25

**Authors:** Xing Chen, Fuyuan Wang, Liquan Lin, Hao Dong, Feifei Huang, Kanhar Ghulam Muhammad, Liying Chen, Olga Y. Gorlova

**Affiliations:** 1 Department of Biomedical Engineering, Key Laboratory of Biomedical Engineering of Ministry of Education of China, Zhejiang University, 38 Zheda Road, Zhou Yi Qing Building, Hangzhou 310027, China; wangfuyuan@zju.edu.cn (F.W.); llqzjlgdx09@163.com (L.L.); eagan_dh@zju.edu.cn (H.D.); kanhargm@zju.edu.cn (K.G.M.); 2 Department of Family Medicine, Sir Run Run Shaw Hospital, School of Medicine, Zhejiang University, 3 Qingchun E Rd, Hangzhou 310016, China; 21418101@zju.edu.cn (F.H.); cly0906@163.com (L.C.); 3 Department of Biomedical Data Science, Geisel School of Medicine at Dartmouth College, One Medical Center Drive, Lebanon, NH 03756, USA; Olga.Y.Gorlova@dartmouth.edu

**Keywords:** lung cancer, volatile organic compounds, smoking, breath test

## Abstract

Lung cancer (LC) screening will be more efficient if it is applied to a well-defined high-risk population. Characteristics including metabolic byproducts may be taken into account to access LC risk more precisely. Breath examination provides a non-invasive method to monitor metabolic byproducts. However, the association between volatile organic compounds (VOCs) in exhaled breath and LC risk or LC risk factors is not studied. Exhaled breath samples from 122 healthy persons, who were given routine annual exam from December 2015 to December 2016, were analyzed using thermal desorption coupled with gas chromatography mass spectrometry (TD-GC-MS). Smoking characteristics, air quality, and other risk factors for lung cancer were collected. Univariate and multivariate analyses were used to evaluate the relationship between VOCs and LC risk factors. 7, 7, 11, and 27 VOCs were correlated with smoking status, smoking intensity, years of smoking, and depth of inhalation, respectively. Exhaled VOCs are related to smoking and might have a potential to evaluate LC risk more precisely. Both an assessment of temporal stability and testing in a prospective study are needed to establish the performance of VOCs such as 2,5-dimethylfuranm and 4-methyloctane as lung cancer risk biomarkers.

## 1. Introduction

Lung cancer has become the most commonly diagnosed cancer as well as the leading cause of cancer death globally [1]. Effective diagnosis of lung cancer at its early stage is still not achieved. Screening is a promising method since survival is improved and mortality is reduced by applying low-dose computed tomography (LDCT) screening to a population at a high risk of lung cancer [2,3]. A model simulation shows that screening will be more efficient for mortality reduction if applied to a precisely defined candidate population [4]. However, there remains a gap of knowledge in the practical implementation of screening on a larger scale [5]. The method of precisely identifying individuals at a high risk of lung cancer is still lacking. The widely used criteria are only based on age and smoking history, and the use of biological characteristics in the existing risk models is limited [6]. Long-term smoking exposure has been shown to permanently change expression of some genes. Volatile organic compounds (VOCs, VOCs in this article refer to VOCs in exhaled breath) might reflect the cumulative smoking exposure and accumulated risk that will finally lead to the initiation of cancer.

In this paper, we proposed a method to identify individuals at a high risk for lung cancer using VOCs pattern in exhaled gas, which was analyzed by thermal desorption coupled with gas chromatography mass spectrometry (TD-GC-MS). As of now, smoking is a strong lung cancer (LC) risk factor and plays an important role in the carcinogenesis of cells in lung and affects the immunological activity and metabolic procedure [7]. How the intensity and duration of smoking will affect the body will be studied by detecting the exhaled VOCs from nonsmokers and smokers with different smoking behaviors. If a potential LC biomarker shows an association with smoking and with LC, its association with LC might be explained purely by smoking. However, a biomarker showing an association with smoking in a healthy (LC free) population might serve as a biomarker of risk. Thus, a study of the association between exhaled metabolic byproducts and risk factors of lung cancer, such as smoking and air pollution, is the necessary first step in evaluating the performance of VOCs as biomarkers and might provide us with a means of estimating LC risk more precisely.

VOCs in exhaled breath are one kind of metabolic byproduct that can be used to monitor health status and the progression of diseases [8,9]. Studies have shown that VOCs can be used to identify lung cancer [10,11,12]. The concentration and category of VOCs are different when the inflammation and cancerization of lungs occur. These changes of metabolic byproducts are generated within or around the inflammatory/cancer cells or due to the immune reaction of the body [13,14]. So far, seven categories of VOCs have been found in exhaled breath that might have the potential to serve as biomarkers of lung cancer: alkanes, alkanol, aldehyde, ketone, lipid, nitrile, and aromatics [15,16,17]. However, it is still unclear if VOCs can represent the pre-stages of carcinogenesis and can be used to identify people at high risk of lung cancer.

The lung carcinogenesis can take years or even decades. Studies provided evidence that tobacco smoking is a major risk factor for lung cancer [18,19]. Cigarette smoking is a mixture of more than 5000 chemical compounds, among which more than 60 are recognized to have a specific carcinogenic potential [7]. The risk of lung cancer increases with the number of cigarettes smoked per day and the years of smoking. For non-smoking people, exposure to secondhand smoke also increases the risk. Our study is meant to propose a novel method to evaluate lung cancer risks before they cause an untreatable disease. These VOCs can be identified to indicate an elevated risk of lung cancer or serve as biomarkers of the earliest stages of lung carcinogenesis.

We used thermal desorption coupled with gas chromatography mass spectrometry (TD-GC-MS) to analyze exhaled breath samples of 122 healthy volunteers who were undergoing a routine annual medical checkup. The exposure to LC risk factors were recorded for all of the subjects. We identified an association study to analyze the relationship of VOCs and risk factors of lung cancer. Our study was undertaken to find VOCs that may serve as markers of risk and may help define a group at high risk of lung cancer more precisely.

## 2. Results

### 2.1. Subjects

**Figure 1:**
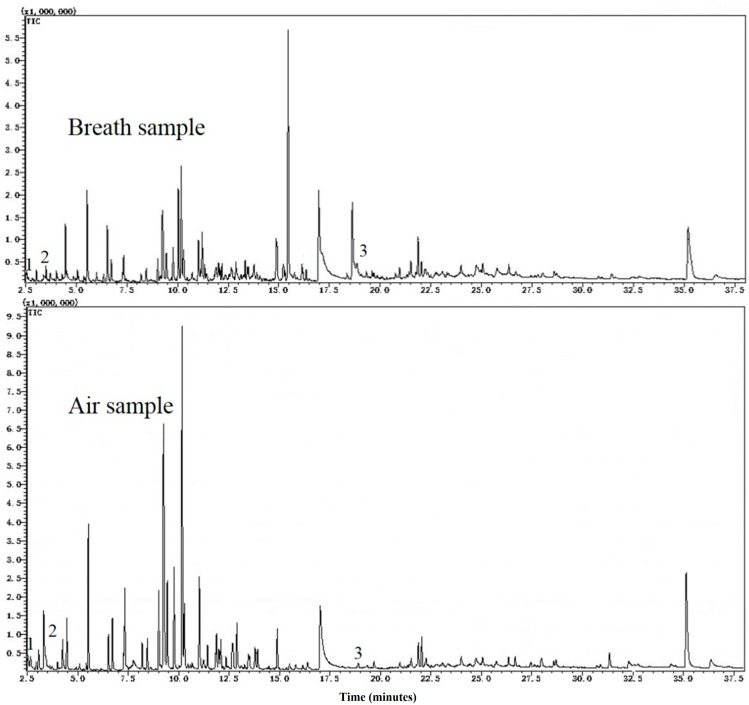
Top of the figure was a sample of exhaled breath, while bottom of the figure was the corresponding air sample. Three substance peaks were marked in the figure, where Peak 1 was *cis*-1,3,5-hexatriene (Chemical Abstracts Service (CAS) register No. 2612-46-6), Peak 2 was 2,5-dimethylfuran (CAS No. 625-86-5), and Peak 3 was decanal (CAS No. 112-31-2).

The characteristics of subjects are presented in Table 1. There were 122 subjects in the study. Male volunteers accounted for 76.2%, while female volunteers accounted for 23.8%. Chromatograms of a breath sample and an ambient air sample are shown in Figure 1. The average age of participants was 49.4 and ranged from 33 to 67. Most participants were aged 40–59. Among our subjects, 59% of the subjects were current smokers, while 34 had never smoked and only 7% were former smokers. Among smokers, 65.4% smoked less than 365 packs per year, 27.2% smoked 365–730 packs per year, and 7.4% smoked more than 730 packs per year. The age of smoking initiation was mainly distributed around 20 years old. The smoking duration was more than 10 years for all ever-smoker participants.

### 2.2. Description of Volatile Organic Compounds (VOCs) Data

**Figure 2:**
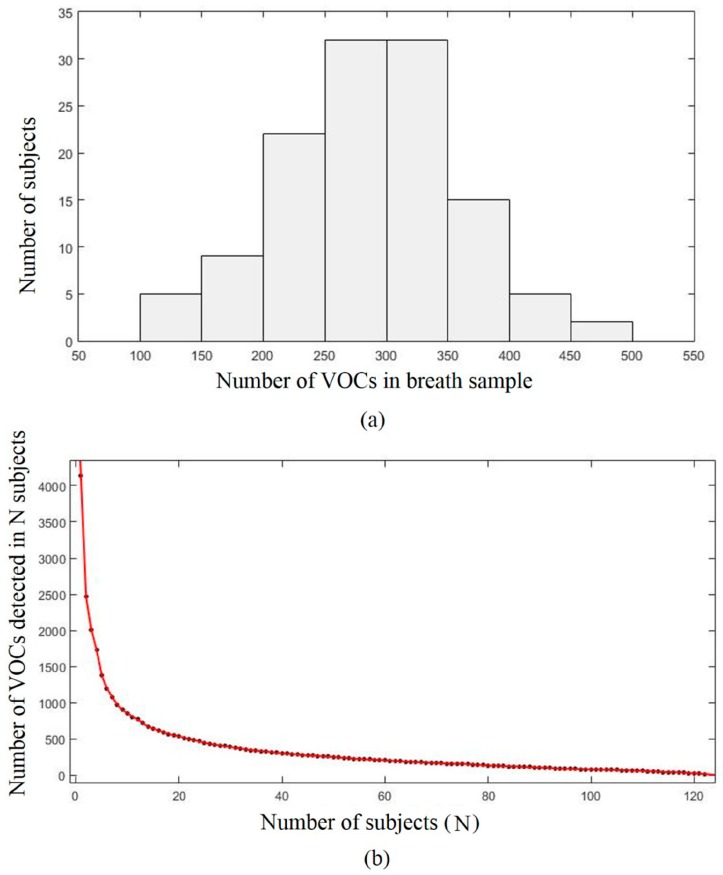
(**a**) The distribution of the number of volatile organic compounds (VOCs) in breath samples; (**b**) The number of VOCs in each subject. The subjects are ranked from the highest to the lowest number of detected VOCs. Overall, 4138 different VOCs were observed in the sample at least once. Only 17 VOCs were observed in all 122 subjects.

The number of VOCs detected in each breath sample ranged from 108 to 452 (mean = 288.3, SD = 71.89) (Figure 2a). The inter-individual variation in the number of detected VOCs displays a normal distribution. The total number of observed VOCs was 4138. Out of 4138 VOCs, 1673 were observed only once, and only 17 VOCs were present in all 122 subjects (Figure 2b). When the subjects were ranked by the number of VOCs detected, the number of VOCs detected in the N-th subject fell rapidly at first and then declined at a slower pace. The number of new identified VOCs presented a downward trend as the number of subjects increased (Figure S1).

### 2.3. The Correlation between VOCs and Smoking

**Figure 3:**
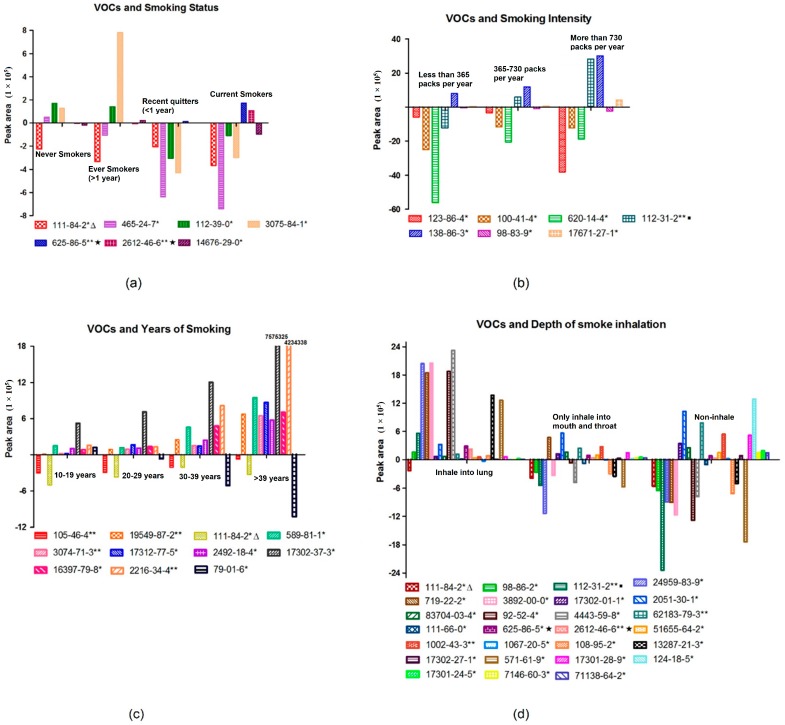
(**a**) VOCs that were correlated with smoking status; (**b**) VOCs that were correlated with smoking intensity; (**c**) VOCs that were correlated with years of smoking; (**d**) VOCs that were correlated with depth of smoke inhalation. (* denotes *p* < 0.05, ** denotes *p* < 0.01; sign ∆ means this compound was correlated with smoking status, years of smoking, and depth of smoke inhalation in the meanwhile, and sign ▪ was both correlated with smoking intensity and depth of smoke inhalation, while sign ★ was both correlated with smoking status and depth of smoke inhalation).

377 VOCs, which were present in more than 30 subjects out of all 122 samples, were observed. The following statistics are based on these 377 VOCs. Results showed that 7, 7, 11, and 27 VOCs were correlated with smoking status, smoking intensity, years of smoking, and the depth of inhalation (Figure 3), respectively. Detailed results such as correlation coefficients are listed in Tables S1–S4. Age and gender were adjusted in the multivariable regression analyses.

**Figure 4:**
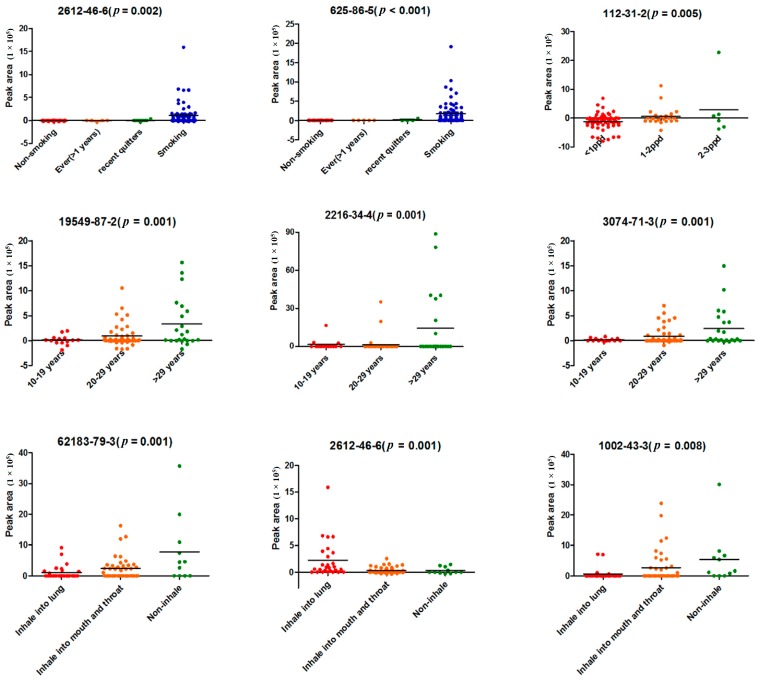
VOCs that were highly significantly correlated with smoking characteristics. (CAS No. 625-86-5, No. 112-31-2, No. 2216-34-4, and No. 62183-79-3 and No. 2612-46-6 remained significant in the correlation with smoking status, smoking intensity, years of smoking, and depth of smoke inhalation, respectively, after adjustment for age and gender).

Several VOCs were highly significantly correlated with those smoking characteristics, and their *p*-values are shown in Figure 4. Among them, Chemical Abstracts Service (CAS) register No. 625-86-5 (2,5-dimethylfuran) and CAS No. 2612-46-6 (*cis*-1,3,5-hexatriene), which were highly significantly correlated with smoking status, were compounds in cigarette gas. CAS No. 112-31-2 (Decanal) was highly significantly correlated with smoking intensity. In addition, 4 and 3 VOCs were highly significantly correlated with years of smoking and the depth of inhalation, respectively. It is worth noting that DL-sec-butyl acetate (CAS No. 105-46-4), which is highly significantly correlated (*p* = 0.006) with years of smoking, was greatly influenced by the ambient air (shown in Figure S2).

## 3. Discussion

Although the average number of VOCs detected in a breath sample was 288.3, which is larger than an average of 204.2 VOCs reported by Michael Phillips in 1999 [9], the difference is not remarkable. The development of GC-MS system and the different definition of chromatographic peak in our method likely account for this difference. It could also reflect the difference between races since the volunteers in our study were all Asian compared to Caucasian and African American subjects in Phillips’ study.

More than 4000 VOCs were observed in 122 subjects. Among them, more than 1600 VOCs were detected only once. In addition, about 200 VOCs were observed in more than 60 subjects, while only 17 VOCs were present in all subjects. There was a considerable inter-individual variation in VOCs. There was no exact same pattern of VOCs in 122 volunteers’ breath. The considerable inter-individual variation was embodied not only in the different number and different kinds of VOCs in each breath sample, but also in the different concentration of VOCs. In the supplementary tables, the standard deviations are very large.

Although a one-time measurement might not be reliable due to limited observations, patterns of VOCs in breath might be used as risk or early disease biomarkers once the temporal variation is evaluated. Furthermore, 12 and 21 VOCs were found that were correlated with gender and age, respectively, pointing out that the exhaled VOCs are related to the change in the activity of metabolic pathways caused by aging and gender difference.

The lung carcinogenesis process usually takes a long time. Cigarette smoking plays an important role in that process, causing a long-term change in the microenvironment and a change in the activity of metabolic pathways of lung cells. Long-term smoking exposure has been shown to permanently change expression of some genes [20]. Smokers also have about a 10-fold higher rate of somatic mutations compared to non-smokers [21]. The change of type and level of metabolic byproducts might reflect the cumulative smoking exposure and accumulated risk that will finally lead to the initiation of cancer. Our study analyzed the relationship between VOCs of healthy people and cigarette smoking to provide data for a better understanding of the change in the microenvironment before the initiation of lung cancer. Recently research has shown a potential usage of microRNAs, which presented in the microenvironment, in the early diagnosis of lung cancer [22]. Associated microRNAs with VOCs might provide information on the change of metabolic pathways, which might reveal the principle of the initiation of lung cancer.

Results showed that both 2,5-dimethylfuran and *cis*-1,3,5-hexatriene existed in cigarette gas and showed a strong correlation with smoking status. 2,5-Dimethylfuran was not detected in non-smokers or former smokers who had stopped smoking for more than 1 year, and *cis*-1,3,5-hexatriene was not detected in non-smokers either. All of the smokers in the study were asked not to smoke for at least 12 h before the sample collection. This meant that the two toxic substances could stay for a longer time in the body than other smoke compounds. The existing time was even longer since those two compounds were also found in the breath of recent quitters who quit smoking for less than a year (Figure 4). In addition, 2,5-dimethylfuran was reported as a biomarker of lung cancer [15]. We had a reason to believe that a long-term exposure to these substances may lead to a certain extent of damage to pulmonary cells or airway epithelial cells. In the other hand, the two compounds were also not observed in some smokers, which may reflect the different metabolism for the two toxic subjects.

Seven and 11 VOCs were related to smoking intensity and years of smoking, respectively. The mean concentration of most VOCs increased as the smoking intensity and duration increased. This shows that the exhale metabolites can reflect the cumulative exposure of smoking. It is worth noting that butyl acetate (*p* = 0.035), which was negatively correlated with smoking intensity in our study, was the only ester reported to be linked to lung cancer [23]. A decreasing concentration of butyl acetate was found in the culture medium with a growing number of lung tumor cells. Tumor cells are more likely to live in a micro-environment with a lower concentration of butyl acetate. Since butyl acetate also decreased as smoking intensity increased, the heavier smokers were more likely to create a micro-environment with a lower concentration of butyl acetate, which is more suitable for the growth of tumor cells. Monitoring butyl acetate in breath might help evaluate the risk of lung cancer in smokers.

In our study, 2,4-dimethyl-1-heptene, DL-sec-butyl acetate, 2,3-dimethylheptane, and 4-methyloctane indicated a strong positive correlation (*p* < 0.01) with years of smoking. 2,4-Dimethyl-1-heptene [17,23] and 4-methyloctane [23,24] were the hydrocarbons reported to be found in the culture medium of lung tumor cells. The concentration of those two VOCs in the culture medium of lung tumor cells was higher than that of the blank medium. Accordingly, those two VOCs also displayed a potential to trace the increasing risk of smoking, although their high concentration in the culture medium of LC cells might reflect the pre-existing smoke exposure of the cells.

Decane, which had a correlation (*p* = 0.031) with the depth of smoke inhalation in our study, was reported as a breath biomarker of lung cancer [12]. The research demonstrated that decane was detected in higher concentrations in both NSCLC and COPD patients compared with healthy volunteers. However, in our study, the concentration of decane was lower in inhaled smoking than non-inhaled smoking. Since decane was only detected in 30 subjects, the limited cases and this pollutant in the background air might have contributed to this observation.

VOC assessment provides a non-invasive approach to identify LC risk factors by evaluation of components of exhaled breath. Breath detection of LC biomarkers, such as butyl acetate, decane, heptane, 2,5-dimethylfuranm and 4-methyloctane, not only can be used to diagnose lung cancer but can also be used to estimate the risk of lung cancer. However, our study has limitations. Although 2,5-dimethylfuranm has a significant *p*-value (<0.05) after Bonferroni correction, the *p*-values are somewhat liberal to most other VOCs. Moreover, our study was only taken in Sir Run Run Shaw Hospital, Zhejiang province, China. This study population might not represent the entire population of China. Though the power of this study was limited, it does suggest that the metabolic VOCs detected in breath may reflect LC risks. Multi-center screening trials are needed to verify the findings. Furthermore, studies have shown not only tobacco smoke but other factors such as asbestos fibers are significant inhaled carcinogens that contribute significantly to lung adenocarcinoma development [25]. In our study, some other risk factors in the questionnaire, such as air pollution and exposures to risk substances, were judged subjectively by participants. As for other risk factors for lung cancer, we focused on “engine oil” and “automobile exhaust.” Although the estimates of the exposure to special substances from the questionnaire were subjective and arbitrary, it is interesting to find that about 50% of related VOCs in the breath were aromatic compounds, which were also components in engine oil and automobile exhaust (Tables S5 and S6). Because we mainly talk about smoking-related markers in the manuscript, the data of other factors were not shown in this paper.

## 4. Materials and Methods

### 4.1. Subjects

We continuously recruited 122 healthy people from December 2015 to December 2016 at Sir Run Run Shaw Hospital, Hangzhou, China. The participants performed exhaled breath test and filled out a questionnaire. People who were undergoing an annual routine examination were asked to participate in the study. The exclusion criteria were as the following: (1) people with an age of detection less than 18 years old; (2) pregnant women; (3) people with other tumor history or suffering from severe infection; (4) people who did not understand or cooperate with any part of the gas collection process. This study was approved by the ethics committee of Sir Run Run Shaw hospital (Approval No. ChiCTR-DCD-15007106, 18 September 2015), and written informed consent was obtained from every participant.

To ensure the accuracy of test results, the participants were required to comply with the following requirements: (1) fasting 12 h before collecting samples; the participants were instructed to avoid high-fat foods at dinner of the evening before the collection; (2) the participants were asked to abstain from smoking for 12 h before collecting samples; (3) antioxidants should not have been used for 24 h prior to the sample collection; (4) gargling was required 15 min before the collection.

Each participant completed a questionnaire. The survey items mainly focused on risk factors of lung cancer. The major risk factor was smoking behavior, so information on smoking status, years of smoking, smoking intensity, the depth of smoke inhalation, and second-hand smoking for participants who never smoked was obtained via the questionnaire.

### 4.2. Breath Collection

The VOCs were captured and concentrated from 1000 mL of exhaled breath into a Tenax TA stainless steel tube (PerkinElmer, Waltham, MA, USA). The capture was realized by a portable breath collection device, which was made in our own lab [26]. The subjects breathed tidally through the mouthpiece assembly, and their breath was stored in a reservoir. The breath reservoir, a heat tube, contained a column of alveolar breath proximal to the outlet valve. The volume of alveolar breath was captured into the sorbent trap through the outlet valve. The process was controlled by varying the flow rate through the flow meter and the duration of sampling. The whole process took about 5 min. These sample tubes were sent to lab for analysis. The sealed Tenax TA stainless steel tube can preserve the exhaled breath sample for a year. The time of shipping among hospital and lab did not affect the results of analysis. Repeated tests on samples of air and human breath were performed to ensure the consistency of the analysis procedures.

### 4.3. Gas Chromatography—Mass Spectrometry-Analysis

The composition analysis procedure was completed via a thermal desorption (TD) instrument (TurboMatrix 300 TD, PerkinElmer, Waltham, MA, USA) and gas chromatography-mass spectrometry (GC-MS) (QP2010 Plus, Shimadzu, Tokyo, Japan). TD was used to desorb the VOCs adsorbed in the Tenax TA stainless steel tube. GS-MS analyses were performed following the desorption procedure. The VOCs, then, were sent into an Rtx-5 column (30.0 m Length × 0.25 mm ID × 0.25 μm Thickness, Restek) to be separated. The carrier gas was helium. The column temperature program was 40 °C initially, held for 1 min, and increased to 250 °C at 5°/min, and held for 1 min at 250 °C. The MS was in scan mode in the 45–500 mass to charge (*m*/*z*) range. The scan time was 2.5–38 min. The temperatures of the electron impact ion source and interface were set at 200 °C and 250 °C, respectively.

### 4.4. Calibration

To verify the stability and reliability of the detection system, a standard gas sample 2,5-dimethylfuranm, which was significantly associated with smoking in our study, was used in calibration experiments. 2,5-Dimethylfuranm (99%, Aladdin, China) was dissolved in methanol (99.5%, Sinopharm chemical Reagent Co., Ltd., Shanghai, China). The mixed solution was converted into mixed gas by a gas generator (MF-3B, developed by China National Metrology Technology Development Co., Beijing, China). The concentrations of 2,5-dimethylfuranm in the mixed gas were 0.1, 0.2, 0.5, 1.0, and 2.0 ppb, which covered the range in exhaled gas. The mixed gas was kept in a 2 L Tedlar bag. The process of analysis was as same as that for exhaled gas. Results indicated the detection system had a good linear response (*R*^2^ = 0.9984, *p* < 0.0001, Figure S3).

### 4.5. Data Analysis

#### 4.5.1. Pretreatment of Volatile Organic Compounds (VOCs)’ Data

VOCs, with a half-height peak width less than 2 s and peak areas larger than 0, were extracted from the chromatogram for each sample. All extracted VOCs were identified according to the mass spectrometry library NIST 05 and NIST 05s. For the extracted VOCs, the values of peak areas in chromatogram were calculated. Data from each extracted VOC, comprising retention time, CAS No., chemical identity, area under the curve (AUC), and quality of fit, were automatically downloaded into a text file.

Ambient air in the room, where the breath test was performed, was also collected as a background for the test. The VOCs in ambient air were also managed according to the procedure described above.

A VOC is not only identified by spectral library match, but also by its retention time [13]. According to the result of identification by the mass spectrometry library NIST 05 and NIST 05s, several VOCs had the same CAS number in the chromatogram. Those VOCs whose time difference of retention time (RT) was less than 0.3 min were regarded as the same compound. We summed their peak areas and averaged their RTs. The calculation process is shown in Equations (1) and (2). A and RT are the final peak area and final RT of a VOC, respectively. $A_{n}$ and $RT_{n}$ are the peak area and retention time of VOCs with the same CAS number, respectively.

Ambient air data as a baseline would be subtracted from the sample data. VOCs presented in breath and in air whose CAS number was the same and the relative time difference of RT was less than 5% were regarded as the same compound. Equation (3) describes the process. Equation (4) shows that the peak area of a VOC in ambient air is subtracted from its corresponding peak area in breath. $\Delta A_{breath}$ is a relative value of a VOC. $A_{breath}$ and $A_{air}$ is the peak area of VOC in breath and in ambient air. The calculations using these four formulas were performed by a computer program.

(1) $$A=A_{1}+A_{2}+\cdot \cdot \cdot +A_{n}$$

(2) $$RT=\frac{RT_{1}+RT_{2}+\cdot \cdot \cdot +RT_{n}}{n}$$

(3) $$\left|\frac{RT_{breath}-RT_{air}}{RT_{breath}}\right|<5\%$$

(4) $$\Delta A_{breath}=A_{breath}-A_{air}$$

#### 4.5.2. Statistical Analysis

VOCs data and data from questionnaire of each volunteer were recorded in a dataset for further analysis. The CAS No., retention time, and peak area of each VOC were included in the dataset. Meanwhile, age, gender, and smoking behavior were also recorded in the dataset. Statistical analysis was done by Statistical Package for the Social Sciences (SPSS) (PASW Statistics 18, IBM Corp., Armonk, NY, USA), GraphPad (Prism 5, GraphPad Software, Inc., La Jolla, CA, USA) and MATLAB (R 2015a, The MathWorks, Inc., Natick, MA, USA) for Windows.

Pearson correlation coefficient was used to screen VOCs that were correlated with risk factors of LC and to evaluate their linear correlations. The t-test was used to compare the correlative VOCs. The level of significance was set to a *p*-value of less than 0.05. A multivariate linear regression analysis was performed to adjust age and gender. Conservative Bonferroni correction was applied to test the significance in multiple analyses.

## 5. Conclusions

In conclusion, we found 7, 7, 11, and 27 VOCs were correlated with smoking status, smoking intensity, years of smoking, and depth of smoke inhalation, respectively. Exhaled VOCs are related to smoking, and might have a potential to evaluate LC risk more precisely. Both an assessment of temporal stability and testing in a prospective study are needed to establish the VOCs’ performance such as 2,5-dimethylfuranm and 4-methyloctane as lung cancer risk biomarkers.

## Figures and Tables

**Table 1:** The characteristics of the study population.

Characteristics	Groups	Study Population (No. = 122)
Gender (*n*, %)	Male	93(76.2)
	Female	29(23.8)
Age (median, range)		49.4(33–67)
Age group (*n*, %)	30–39	11(9.1)
	40–49	50(41.3)
	50–59	50(41.3)
	60–69	10(8.3)
Smoking status (*n*, %)	Current	72(59.0)
	Former	9(7.4)
	Never	41(33.6)
Smoking intensity (mean ± SD, Median) (*n*, %) ^(CF)^	<365 packs/year (245 ± 126, 273)	53(65.4)
	365–730 packs/year (578 ± 96, 548)	22(27.2)
	>730 packs/year (1095 ± 365, 912)	6(7.4)
Age at starting smoking (*n*, %) ^(CF)^	Less than 15	2(2.5)
	15–19	18(22.2)
	20–24	39(48.1)
	25–29	13(16.1)
	More than 29	9(11.1)
Years of smoking (*n*, %) ^(CF)^	10–19 years	14(17.3)
	20–29 years	45(55.6)
	>29 years	22(27.1)

^(CF)^: limited to current and former smokers.

## Supplementary Materials

Supplementary materials can be found at www.mdpi.com/1422-0067/18/11/2235/s1.
