# Supplementary material for: Association of Smoking with Metabolic Volatile Organic Compounds in Exhaled Breath
Source: Int J Mol Sci. 2017 Oct 25;18(11):2235. doi: 10.3390/ijms18112235 (PMC5713205; doi:10.3390/ijms18112235)
Supplement: Supplementary file 1 [file ijms-18-02235-s001.pdf]

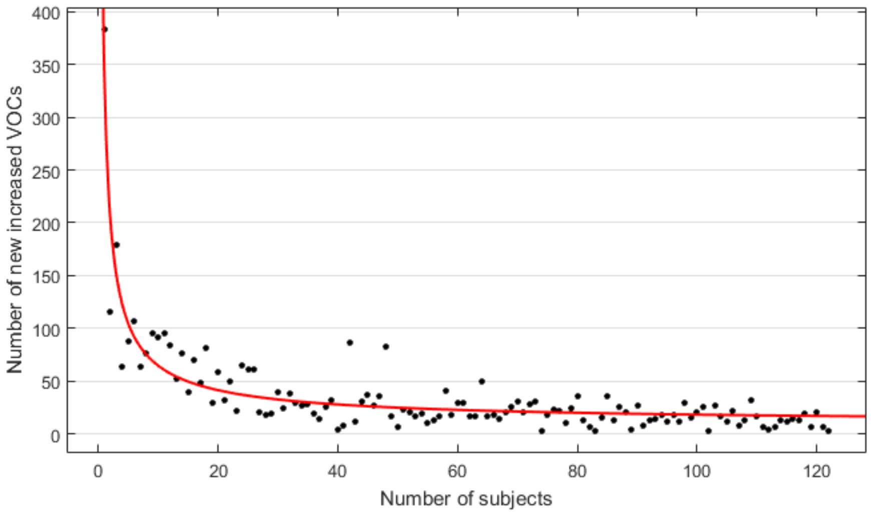

Figure S1. A downward trend of number of new identified VOCs.

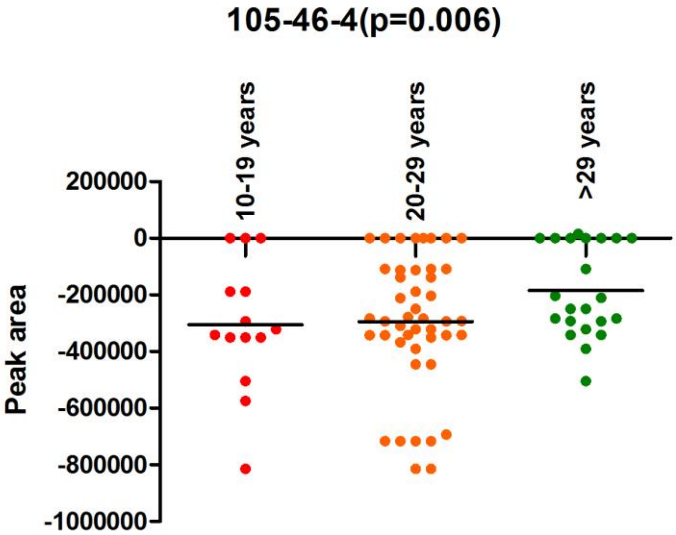

Figure S2. The VOC was strong correlated with years of smoking.

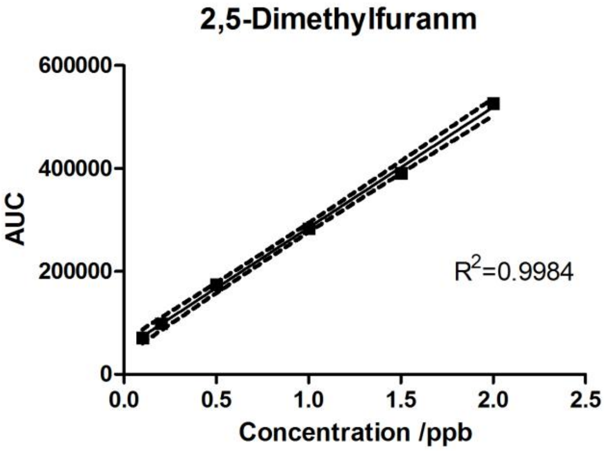

Figure S3. The results of calibration.

Table S1. The means of VOCs associated with smoking status.

CAS No.	VOCs	SS	Number of Participants	*p*-Value
**111-84-2 ***	Nonane	1	41	0.047
		2	5	
		3	4	
		4	72	
**465-24-7 ***	Longiborneol	1	41	0.022
		2	5	
		3	4	
		4	72	
**112-39-0 ***	Methyl hexadecanoate	1	41	0.033
		2	5	
		3	4	
		4	72	
**3075-84-1 ***	2,2′,5,5′-Tetramethylbiphenyl	1	41	0.04
		2	5	
		3	4	
		4	72	
**625-86-5 ****	2,5-Dimethylfuran	1	41	<0.001
		2	5	
		3	4	
		4	72	
**2612-46-6 ****	*cis*-1,3,5-Hexatriene	1	41	0.002
		2	5	
		3	4	
		4	72	
**14676-29-0 ***	3-Ethyl-2-methylheptane	1	41	0.032
		2	5	
		3	4	
		4	72	

* $p < 0.05$, ** $p < 0.01$. SS: smoking status: never, former, former that quit less than 1 year, and current are signed as 1, 2, 3, and 4, respectively.

Table S2. The means of VOCs associated with smoking intensity.

CAS No.	VOCs	SI	Number of Participants	*p*-Value
**123-86-4 ***	Butyl acetate	1	53	0.035
		2	22	
		3	6	
**100-41-4 ***	Ethylbenzene	1	53	0.043
		2	22	
		3	6	
**620-14-4 ***	3-Ethyltoluene	1	53	0.046
		2	22	
		3	6	
**112-31-2 ****	Decanal	1	53	0.005
		2	22	
		3	6	
**138-86-3 ***	Cinene	1	53	0.026
		2	22	
		3	6	
**98-83-9 ***	2-Phenyl-1-propene	1	53	0.026
		2	22	
		3	6	
**17671-27-1 ***	Behenyl behenate	1	53	0.04
		2	22	
		3	6	

* $p < 0.05$, ** $p < 0.01$. SI: smoking intensity. 1: 1–20 cigarettes per day, 2: 21–40 cigarettes per day, 3: 41–60 cigarettes per day.

Table S3. The means of VOCs associated with years of smoking.

CAS No.	VOCs	YS	Number of Participants	*p*-Value
**111-84-2 ***	N-Nonane	1	14	0.028
		2	45	
		3	22	
**19549-87-2 ****	2,4-Dimethyl-1-heptene	1	14	0.001
		2	45	
		3	22	
**105-46-4 ****	DL-sec-Butyl acetate	1	14	0.006
		2	45	
		3	22	
**589-81-1 ***	3-Methylheptane	1	14	0.014
		2	45	
		3	22	
**3074-71-3 ****	2,3-Dimethylheptane	1	14	0.001
		2	45	
		3	22	
**17312-77-5 ***	2,3-dimethylundecane	1	14	0.013
		2	45	
		3	22	
**2492-18-4 ***	Hexanoic acid, 1,1-dimethylethyl ester	1	14	0.049
		2	45	
		3	22	
**17302-37-3 ***	2,2-Dimethyldecane	1	14	0.011
		2	45	
		3	22	
**16397-79-8 ***	Acetic acid,2,2,2-trichloro-, 2-ethylhexyl ester	1	14	0.01
		2	45	
		3	22	
**2216-34-4 ****	4-Methyloctane	1	14	0.001
		2	45	
		3	22	
**79-01-6 ***	Trichloroethylene	1	14	0.021
		2	45	
		3	22	

* $p < 0.05$, ** $p < 0.01$. YS: years of smoking years of smoking. 1: 10–19 years, 2: 20–29 years, 3: >29 years.

Table S4. The means of VOCs associated with depth of smoke inhalation.

CAS No.	VOCs	DS	Number of Participants	*p*-Value
**111-84-2 ***	n-Nonane	1	27	0.013
		2	42	
		3	11	
**98-86-2 ***	Acetophenone	1	27	0.027
		2	42	
		3	11	
**112-31-2 ***	Decyl aldehyde	1	27	0.042
		2	42	
		3	11	
**24959-83-9 ***	2,4-Methano-1H-indene,octahydro-7a-methyl-1-methylene-5-(1-methylethyl)-, (2R,3aR,4R,5S,7aS)-rel-	1	27	0.042
		2	42	
		3	11	
**719-22-2 ***	2,6-Di-tert-butylcyclohexa-2,5-diene-1,4-dione	1	27	0.042
		2	42	
		3	11	
**3892-00-0 ***	2,6,10-Trimethylpentadecane	1	27	0.049
		2	42	
		3	11	
**17302-01-1 ***	3-Ethyl-3-methylheptane	1	27	0.049
		2	42	
		3	11	
**2051-30-1 ***	2,6-Dimethyloctane	1	27	0.033
		2	42	
		3	11	
**83704-03-4 ***	Heptane,3-[(1,1-dimethylethoxy)methyl]-	1	27	0.02
		2	42	
		3	11	
**92-52-4 ***	Biphenyl	1	27	0.027
		2	42	
		3	11	
**4443-59-8 ***	5-Cyclohexylicosane	1	27	0.044
		2	42	
		3	11	
**62183-79-3 ****	2,2,4,4-Tetramethyloctane	1	27	0.001
		2	42	
		3	11	
**111-66-0 ***	1-Octene	1	27	0.02
		2	42	
		3	11	
**625-86-5 ***	2,5-Dimethylfuran	1	27	0.01
		2	42	
		3	11	
**2612-46-6 ****	1,3,5-Hexatriene, (3Z)-	1	27	0.001
		2	42	

		3	11	
**51655-64-2 ***	3-Methylene-nonane	1	27	0.033
		2	42	
		3	11	
**1002-43-3 ****	3-Methylundecane	1	27	0.008
		2	42	
		3	11	
**1067-20-5 ***	3,3-Diethylpentane	1	27	0.014
		2	42	
		3	11	
**108-95-2 ***	Phenol	1	27	0.02
		2	42	
		3	11	
**13287-21-3 ***	6-Methyltridecane	1	27	0.043
		2	42	
		3	11	
**17302-27-1 ***	2,5-Dimethylnonane	1	27	0.037
		2	42	
		3	11	
**571-61-9 ***	1,5-Dimethylnaphthalene	1	27	0.049
		2	42	
		3	11	
**17301-28-9 ***	3,6-Dimethylundecane	1	27	0.033
		2	42	
		3	11	
**124-18-5 ***	Decane	1	27	0.031
		2	42	
		3	11	
**7146-60-3 ***	2,3-Dimethyloctane	1	27	0.015
		2	42	
		3	11	
**17301-24-5 ***	2,7-Dimethylundecane	1	27	0.033
		2	42	
		3	11	
**71138-64-2 ***	3-Methyleneundecane	1	27	0.03
		2	42	
		3	11	

* $p < 0.05$, ** $p < 0.01$. DS: depth of smoke inhalation. 1: inhale into lung, 2: only inhale into mouth, and throat, 3: non-inhale.

Table S5. Correlations between VOCs and "engine oil" reported.

CAS No.	VOCs	*p*-Value
**95-47-6**	1,2-Dimethylbenzene	0.042
**1120-21-4**	n-Hendecane	0.004
**620-14-4**	1-ethyl-3-methyl-Benzene	0.028
**294-62-2**	Cyclododecane	<0.001
**77-53-2**	Cedrol	<0.001
**123-51-3**	1-Hydroxy-3-methylbutane	0.014
**2958-76-1**	Naphthalene,decahydro-2-methyl-	<0.001

Table S6. Correlations between VOCs and "automobile exhaust" reported.

CAS No.	VOCs	*p*-Value
**95-47-6**	1,2-Dimethylbenzene	0.030
**13475-82-6**	2,2,4,6,6-pentamethyl-Heptane	<0.001
**80-56-8**	2,6,6-Trimethylbicyclo[3.1.1]hept-2-ene	0.013
**88-18-6**	2-tert-Butyl-1-hydroxybenzene	0.058
**535-77-3**	1-methyl-3-(1-methylethyl)-benzen	0.023
**2958-76-1**	Naphthalene,decahydro-2-methyl-	0.031
